# A reconstruction of parasite burden reveals one century of climate-associated parasite decline

**DOI:** 10.1073/pnas.2211903120

**Published:** 2023-01-09

**Authors:** Chelsea L. Wood, Rachel L. Welicky, Whitney C. Preisser, Katie L. Leslie, Natalie Mastick, Correigh Greene, Katherine P. Maslenikov, Luke Tornabene, John M. Kinsella, Timothy E. Essington

**Affiliations:** ^a^School of Aquatic and Fishery Sciences, University of Washington, Seattle, WA 98105; ^b^Department of Arts and Sciences, Neumann University, Aston, PA 19014; ^c^Unit for Environmental Sciences and Management, North–West University, Potchefstroom 19014, South Africa; ^d^Department of Ecology, Evolution, and Organismal Biology, Kennesaw State University, Kennesaw, GA 30144; ^e^Northwest Fisheries Science Center, National Oceanic and Atmospheric Administration, Seattle, WA 98112; ^f^Burke Museum of Natural History and Culture, Seattle, WA 98195; ^g^HelmWest Laboratory, Missoula, MT 59801

**Keywords:** biological collections, infectious disease, environmental change, historical ecology, museum specimens

## Abstract

Long-term data allow ecologists to assess trajectories of population abundance. Without this context, it is impossible to know whether a taxon is thriving or declining to extinction. For parasites of wildlife, there are few long-term data—a gap that creates an impediment to managing parasite biodiversity and infectious threats in a changing world. We produced a century-scale time series of metazoan parasite abundance and used it to test whether parasitism is changing in Puget Sound, United States, and, if so, why. We performed parasitological dissection of fluid-preserved specimens held in natural history collections for eight fish species collected between 1880 and 2019. We found that parasite taxa using three or more obligately required host species—a group that comprised 52% of the parasite taxa we detected—declined in abundance at a rate of 10.9% per decade, whereas no change in abundance was detected for parasites using one or two obligately required host species. We tested several potential mechanisms for the decline in 3+-host parasites and found that parasite abundance was negatively correlated with sea surface temperature, diminishing at a rate of 38% for every 1 °C increase. Although the temperature effect was strong, it did not explain all variability in parasite burden, suggesting that other factors may also have contributed to the long-term declines we observed. These data document one century of climate-associated parasite decline in Puget Sound—a massive loss of biodiversity, undetected until now.

## Significance

Parasites are influential in ecosystems, both for better (e.g., facilitating energy flow through food webs) and for worse (e.g., compromising the conservation status of host species). Unfortunately, there are few data to indicate how parasite populations are changing through time. We extracted data on metazoan parasite abundance from marine fish specimens held in natural history collections, finding one century of decline in abundance for some groups of parasites with complex life cycles. This decline was correlated with increases in sea surface temperature, suggesting a climate change-associated loss of parasite biodiversity. While a decline in parasitism might sound like a stroke of good luck, vital ecological functions could be lost alongside parasites.

After many decades of being ignored in ecological research (1–2), parasites (3, 4) are increasingly recognized as influential players in ecosystems (5)—both for their positive effects on ecosystem function and for their negative effects on valued host species. Through their ability to regulate host populations (6), parasites can threaten the conservation status of rare hosts (7), and this has provoked justifiable concern that increasing parasite abundance could jeopardize conservation successes. But although they can have negative fitness impacts on their hosts, parasites also perform essential ecological functions at the community and ecosystem levels by providing population regulation for hosts that would otherwise become overabundant, facilitating the flow of biomass, and increasing food web connectivity (5, 8).

For many other groups of organisms, ecologists rely on long-term data to infer current population status and trends and to project future threats: for example, some insects (9), amphibians (10), mammals (11), and birds (12) are on the decline, whereas invasive species (13) and synanthropic species (14) are on the rise. Given their substantial ecological influence, it would be useful to have similar long-term data for parasites (15), but almost no such data exist for parasites of nonhuman hosts (16, 17). Those few datasets that do exist are plagued with issues of accessibility, completeness, temporal scope, and taxonomic coverage (17, 18). To reveal whether parasites represent a rising threat to conservation or a collateral impact of environmental change (or both), nothing will substitute for long-term data across many parasite species.

There is good reason to expect that parasite abundances have changed through time, although few clear and convincing directional predictions have emerged. For example, rising temperatures associated with climate change could affect temperature-dependent parasite vital rates directly, affect transmission success by altering host immune defenses, or change host density, which may have positive, negative, or neutral net effects on parasite abundance (19–20). Pollutants can erode host immune defenses, increasing transmission, but may also cause direct mortality of parasite infectious stages, decreasing transmission (21–24). Exploitation of natural resources could decrease host density, which should reduce transmission (25, 26), but it can also cause compensatory increases in nontarget species, increasing transmission for parasites of those hosts (27–28). These predictions are further complicated by variation among the parasites; directly transmitted parasites (i.e., parasites that can be transmitted between conspecific hosts) are expected to have more positive responses to global change than are complex life cycle parasites (i.e., parasites that require multiple host species to complete their life cycles) (27–28), and each additional obligately required host in a parasite life cycle is expected to add more vulnerability (29, 30). Crosscurrents of global anthropogenic change manifest simultaneously in ecosystems; the only way to detect their net effect on parasites is to actually measure how parasite populations change through time.

Luckily, there does exist a technique for extracting temporally resolved, long-term data on parasites of the past: Specimens held in natural history collections are often fixed and stored in such a way that both host and parasite tissues are maintained (15, 18, 31). Parasitological dissection, visual detection, and morphological identification can therefore yield accurate counts of the number and identity of metazoan parasites infecting a host at the time of its death (18).

**Fig. 1.**
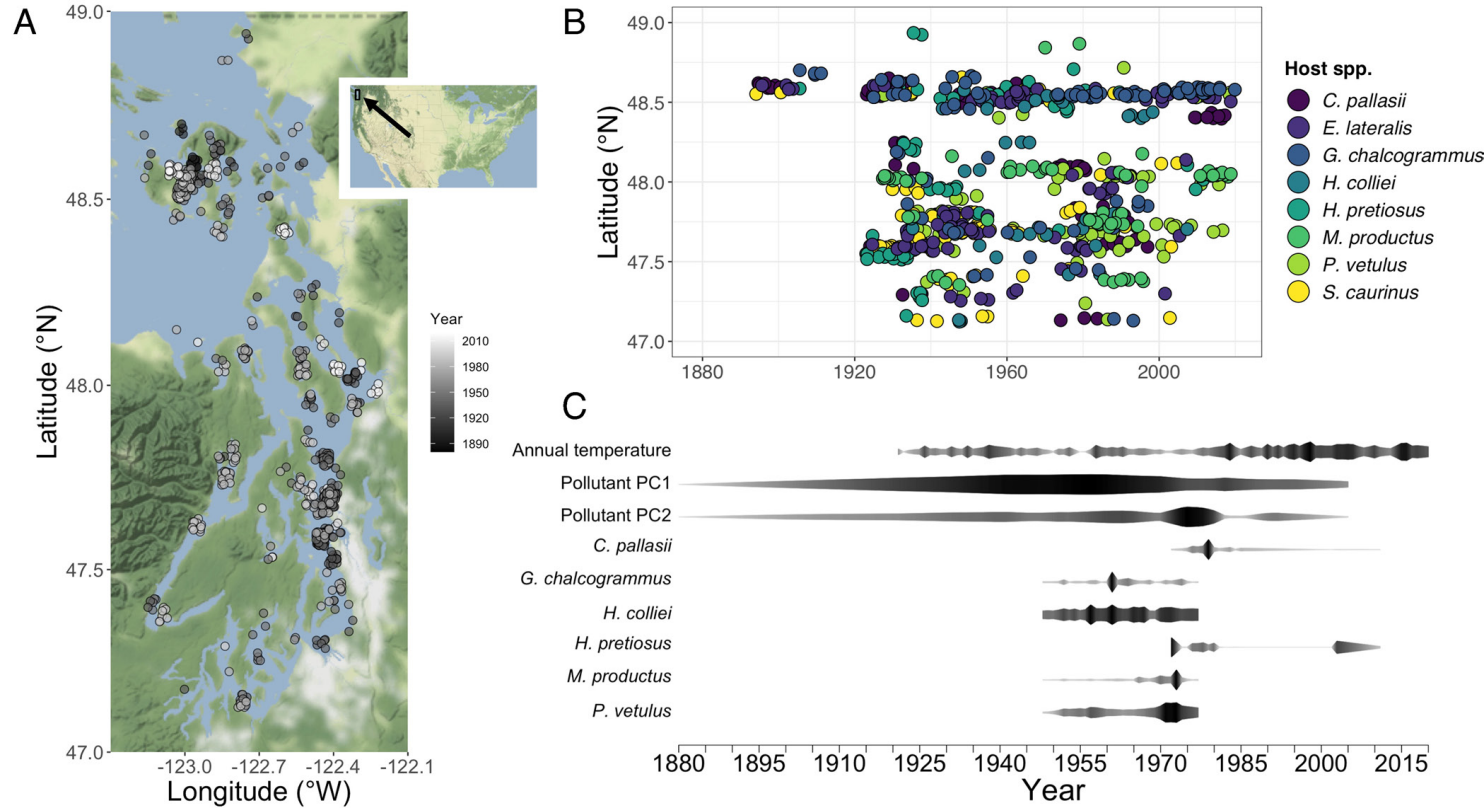
(*A*) Map of sampling location and year of collection (darker color = older) for each fish whose parasite burden was evaluated. Black rectangle in the *Inset* shows boundaries of the map. (*B*) Distribution through time (x axis) and across latitude (y axis) of fish sampled within each host species. Note that a few lots missing location information (e.g., the earliest lots from 1880) are not plotted here (*Materials and Methods*). (*C*) Historical timeline for potential environmental drivers of change in parasite burden. The width and darkness of each band is proportional to the intensity of each driver (each scaled relative to itself). Annual temperature data from ref. 34, pollutant data from ref. 35, data on abundance of *G. chalcogrammus*, *H. colliei*, *M. productus*, and *P. vetulus* from ref. 36, and data on abundance of *C. pallasii* and *H. pretiosus* from ref. 37.

We used this technique to generate a well-resolved, long-term time series of parasite abundance for a multihost parasite assemblage. We reconstructed approximately one century (1880 to 2019, but the vast majority of data span 1920 to 2019) of change in parasite abundance (i.e., parasite counts per host individual) for eight fish hosts (*SI Appendix*, Tables S1 and S2) and 85 common parasite taxa (*SI Appendix*, Table S3) in Puget Sound, Washington. Puget Sound is the second-largest estuary complex in the conterminous United States (32) and an ecosystem that has experienced substantial environmental change over the past century (Fig. 1), including a 1 °C increase in sea surface temperature (SST) between 1950 and 2005 (33). Our findings reveal a broad-scale decline in metazoan parasite abundance that spans parasite taxonomic groups, suggesting that the contemporary Puget Sound ecosystem may be impoverished in parasites compared with its historical state.

## Results

We sourced fluid-preserved fish specimens from natural history collections across North America and performed a modified parasitological dissection that minimizes damage to these irreplaceable specimens while maximizing our ability to visually detect parasites (18, 38). For each host species–decade combination between 1920 and 2020, we dissected a median of 9.5 fish individuals (range = 0 to 20; *SI Appendix*, Table S2). A few additional fish were available between 1880 and 1920 (*SI Appendix*, Table S2). For each specimen examined, we noted locality data from natural history collection databases and record cards stored with the specimens and measured the host’s total length (TL) in centimeters. We identified all parasites to the lowest possible taxonomic level. Our primary statistical framework for testing hypotheses and evaluating effect sizes was to fit hierarchical models to counts of the number of individuals of each parasite taxon in each fish specimen. We aimed to control for potential confounding effects caused by differences in individual fish hosts, the location of capture, and the size of the fish host, as well as how those effects varied among each host–parasite combination. We then tested whether, after accounting for those effects, there was evidence of association between parasite abundance and environmental covariates of interest, including indices of temperature, pollution, and host density.

We counted a total of 17,702 parasites from 699 fish specimens. To isolate parasite taxa that were sufficiently abundant for analysis, we selected those taxa that were found in at least 5% of the host individuals dissected within a host species and across all years (i.e., parasites that occurred at 5% prevalence in their host species across all years). This yielded 85 parasite taxa (20 directly transmitted parasites with a single host, 21 complex life cycle parasites with two hosts, and 44 complex life cycle parasites with three or more hosts; n = 17,259 individuals across these 85 common taxa).

**Fig. 2.**
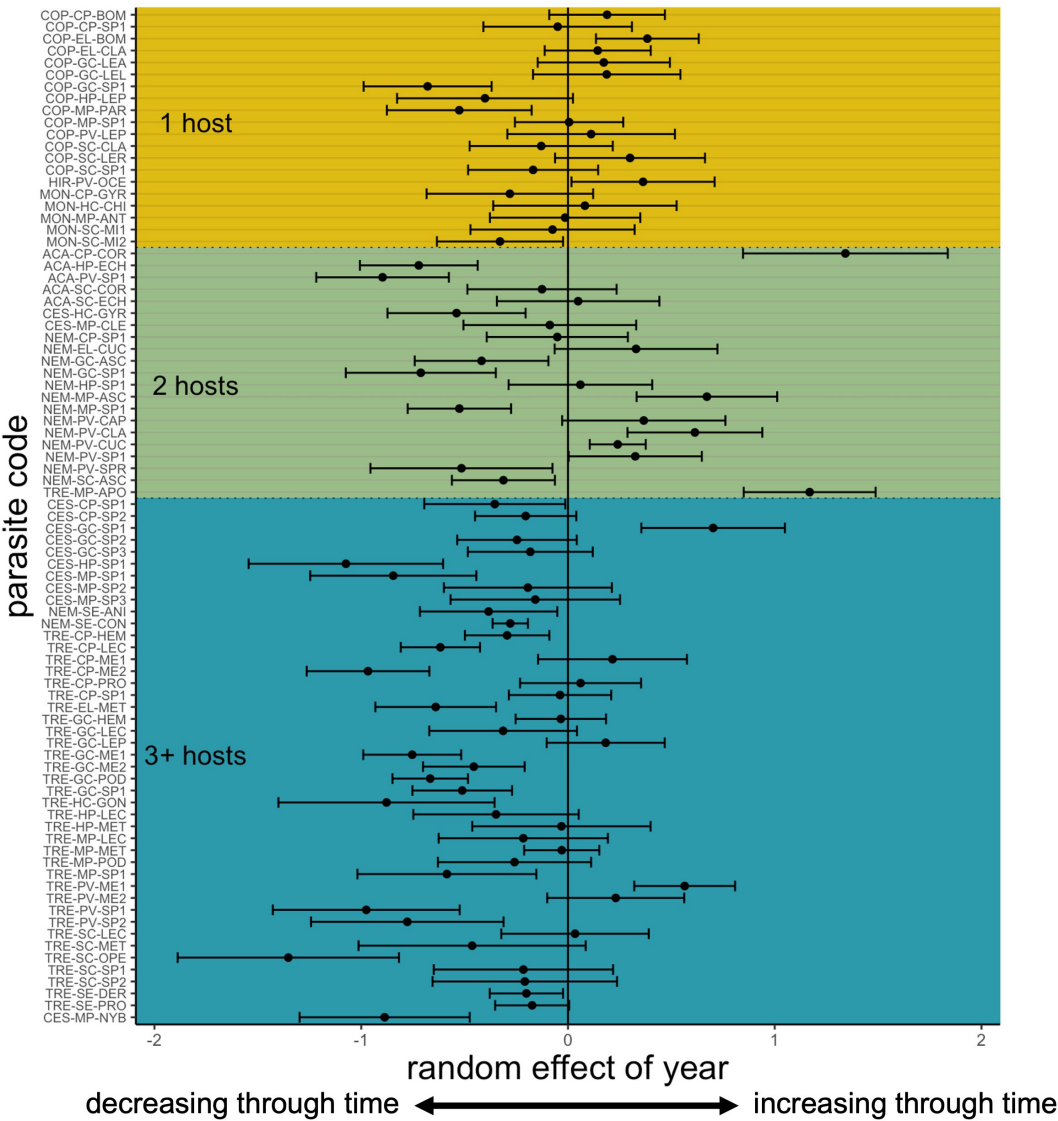
Estimated effect of year for each parasite taxon extracted from the generalized linear model that described change over time in parasite abundance (*Materials and Methods* and Eq. **3**). Parasite codes are related to taxon names in *SI Appendix*, Table S3. The x axis is the random effect of year on the abundance of each parasite taxon, and error bars represent SE on each random effect.

**Fig. 3.**
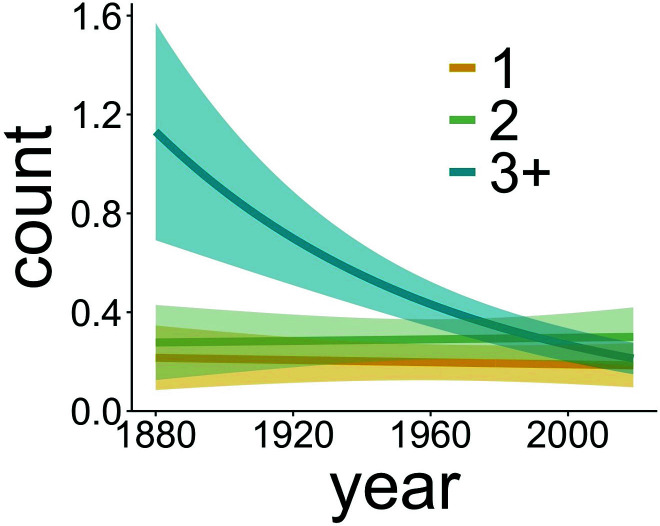
The count per host of parasites that obligately require three or more hosts declined through time, while that of two- and one-host parasites remained stable. Shown are model predictions between the years 1880 and 2019 from phase 1 analysis. Predictions are for the “average” parasite species within each group (i.e., within 1-host parasites, 2-host parasites, and 3+-host parasites) where average is defined as “the parasite species with the abundance that is closest to the average abundance of all parasite species.”

The first phase of analysis provided overwhelming support for models that included an effect of time on parasite counts (details of model selection are provided in *SI Appendix*, Table S4) as the model lacking a year effect had essentially zero support (ΔAIC = 62.5). The best model included parasite taxon-specific effects of year grouped by the number of obligately required host species in the parasite life cycle (Fig. 2). This model showed that parasite taxa with life cycles requiring three or more hosts exhibited a steep decline in abundance over time (10.9% per decade), whereas taxa with one or two hosts showed little change over time (Fig. 3). The model that had parasite taxon-specific year effects with no additional grouping had less support (ΔAIC = 1.12). There was also less support for the model containing year effects grouped by broad taxonomic classifications (i.e., Subclass Copepoda, Subclass Hirudinea, Class Monogenea, Class Trematoda, Class Cestoda, Phylum Nematoda, and Class Acanthocephala; ΔAIC = 3.32).

We noted that 10 parasite taxa were present—sometimes at high (>20%) prevalence—in older fish specimens but were absent from more recent specimens. All were too rare in our dataset (i.e., had too few observations) to allow a formal analysis, but we identified those parasite taxa not observed in the past ≥40 y and plotted their frequency of occurrence to highlight the possibility of recent local extirpations (*SI Appendix*, Fig. S1). Among 10 parasite taxa not observed in the past ≥40 y, nine were complex life cycle parasites; among these nine complex life cycle parasites, eight required three hosts and one required four hosts. Complex life cycle parasites with three or more hosts were therefore disproportionately represented among parasites that were absent in the last few decades of the dataset.

**Fig. 4.**
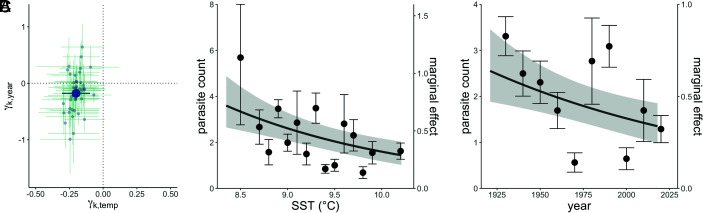
(*A*) Estimated effects (±SE) of temperature (x axis) and year (y axis) on parasite counts for each parasite taxon (light blue), with mean effect size (±SE) over all taxa (dark blue) that have three or more obligately required hosts. (*B*) Parasite count as a function of SST in degrees Celsius in the year of the host’s collection (left axis, points) and fitted relative density of predictions (right axis, line). Points are parasite counts averaged within 0.1-degree increments; increments with fewer than 25 observations were excluded from this plot. (*C*) Parasite count as a function of year of the host’s collection (left axis, points) and fitted relative density of predictions (right axis, line). Points are parasite counts averaged within 10-y increments; increments with fewer than 25 observations were excluded from this plot. For both (*B*) and (*C*), the line represents predictions from a generalized linear mixed model, and the ribbon indicates ±1 SE of prediction. Predictions are for an average parasite taxon of an average host–parasite pair. Because of differences in baseline prevalence and counts when present, the prediction line for an average parasite taxon and average host–parasite pair (the marginal effect) will not equal the averages across data bins.

In the second phase of analysis, we analyzed only data for parasites with three or more obligately required hosts because the first phase revealed that these taxa exhibited changes in abundance over time. By fitting the statistical model using different combinations of environmental variables in place of or in addition to year, we identified which hypothesized predictor variables were supported by the data. Of the three environmental variables examined (i.e., SST, pollutants, and host density), only temperature could explain the overall change in parasite counts (*SI Appendix*, Table S5); the best-fitting model included both annual temperature and year as predictor variables. Notably, parasite taxon responses to increasing temperature were extremely consistent among taxa and were always negative (Fig. 4*A*). In comparison, the parasite taxon-level year trends were far more variable, ranging from −1.0 to +0.64 (Fig. 4*A*), which suggests that other latent drivers have important but highly taxon-specific effects on parasite counts.

While this phase of analysis also revealed that parasite counts were associated with pollutant values (*SI Appendix*, Table S5), the mean effect of pollutants across parasite taxa was near 0, with high variability in individual parasite taxon responses (*SI Appendix*, Fig. S2). Essentially, pollutants impacted the abundance of some individual parasite taxa, but the direction and magnitude of this effect varied across parasite taxa, and pollution was therefore an unlikely explanation for the wholesale declines observed in phase 1 of analysis.

The effect of host density was estimated with low precision, owing to the shorter duration of the time series (*SI Appendix*, Table S5). The fact that host density was not a predictor of parasite counts could arise from these gaps in the time series of host density data but may also hint that the host species we dissected are incidental in the life cycles of the parasites detected or at least that they do not generally represent life cycle bottlenecks for those parasites (29).

## Discussion

Many have hypothesized that climate change may imperil parasite species (8, 29, 30, 39–40), but our dataset provides the first empirical evidence for this effect. We found that parasite taxa obligately requiring three or more hosts to complete their life cycles (i.e., 44 of 85 or 52% of all parasite taxa detected) declined in abundance at a rate of 10.9% per decade or by 38% for every 1 °C increase in SST. We found no significant change in abundance over time among the parasite taxa that require one or two hosts. The effect sizes of time and temperature on the abundance of parasites with three or more hosts were nearly identical (Fig. 4 *B* and *C* and *SI Appendix*, Table S5), indicating that annual SST dynamics account for roughly one-half of the observed dynamics in parasite abundance. Parasite responses to SST were remarkably consistent among parasite taxa (Fig. 4*A*), suggesting that temperature change is a dominant mechanism underlying the effect of time on the abundance of these parasites. However, a considerable portion of the decline in parasite counts remains unexplained, indicating that there are unobserved drivers causing time dynamics (Fig. 4*B*).

The association between climate and parasite burden is merely correlative, and as a correlative association, it could be spurious. It is possible that some other time-associated driver is the true cause of declines among 3+-host parasites, and SST is correlated with parasite burden merely because it is also associated with time. Other variables that might be the “true” driver of declining parasite burdens (41, 42) include ocean pH (43), hypoxia (44), or harmful algal blooms (45). [Note that analysis of historical fish density has revealed only a few fish species exhibiting monotonic trends over the late 20th century (36, 37)]. These other monotonically changing variables could not be included in our analysis because we could identify no long-term dataset that recorded or hindcasted change in these variables over the period of interest. However, since climate change is a key driver of each of these other monotonically changing variables (41, 42), we conclude that – although we are unsure whether temperature per se drives declines in 3+-host parasites – we suspect that, whatever the driver, it is associated with a changing climate.

Why would climate change diminish parasites with three or more obligately required hosts but not parasites with two or fewer hosts? We propose two potential mechanisms that are not mutually exclusive: the “winners and losers” hypothesis and the “phenological mismatch” hypothesis.

The “winners and losers” hypothesis assumes that parasite life histories must contain a rate-limiting step, which regulates the rate of transmission and therefore of population increase (29). Climate change might influence the pace at which that rate-limiting step proceeds because it could cause some host species to increase in abundance (“winners”) while others decline (“losers”) (46–47). If a complex life cycle parasite is using a “winner” host, it can only benefit to a certain extent until it becomes limited by the availability of another host in the cycle (i.e., until some other host becomes the rate-limiting step). But if a parasite is using a “loser” host, a decline in that host can substantially reduce parasite transmission and even drive that parasite locally extinct. There is therefore limited upside and unlimited downside for complex life cycle parasites exposed to changing conditions.

Parasites with three or more hosts should be especially sensitive to such environmental change because their life cycles can collapse with the loss of any one obligately required host species, and with more hosts come more potential failure points (27, 29, 30). In fact, all of the 3- and 4-host parasites in our dataset use at least two vertebrate hosts, whereas the 2-host parasites use only one; given that marine vertebrates are especially sensitive to climate change (48, 49), this may make 3+-host parasites uniquely vulnerable. In Puget Sound, there are many potential failure points for complex life cycle parasites: long-term declines have been documented among benthic invertebrates (50), marine birds (51), salmon (52), other fishes (36, 37), and some marine mammals (53, 54); some of these shifts have been attributed to climate change, including declines of the intertidal clam *Leukoma staminea* (50) and of Pacific cod (*Gadus macrocephalus*) (36, 55, 56). We did not detect a relationship between host density and parasite abundance, at least for the six host species from which parasites were dissected and where we had sufficient historical data (Fig. 1*C*); however, the “winners and losers” hypothesis refers to a broader suite of hosts than these six, extending to all obligately required hosts in the life cycle, be they invertebrates, fishes, mammals, or birds. It is possible for parasite species to vary in which host species or even life cycle stage constitutes the rate-limiting-step, but what all of these parasites have in common is that their life cycles are playing out on a template of shifting conditions. These climate-driven changes are expected to introduce transmission bottlenecks wherever a parasite is unlucky enough to require a host that is “losing.”

The “phenological mismatch” hypothesis posits that as SSTs rise, host and parasite phenology can become decoupled, resulting in lost transmission opportunities and consequent reductions in parasite abundance. Warming-driven host–parasite decoupling has been reported among trematode parasites of amphibians (57, 58), brood parasites of avian hosts (59), and nematode parasites of livestock (60). In Puget Sound, climate-driven phenological shifts are occurring among several potential hosts, including zooplankton (61, 62), benthic invertebrates (63), and salmon (64, 65). Such climate-driven temporal mismatching could produce the declines we observed among 3+-host parasites—declines that we would expect to observe primarily among those parasites with multiple host species whose phenology must align with that of the parasite for successful transmission.

One alternative explanation for the observed pattern could be physiology—that increasing temperatures could act directly on parasite vital rates—for example by slowing parasite development (66, 30). However, if anything, we would expect climate to exert these effects on directly transmitted parasites, since their transmission from host to host tends to be less buffered by the internal environment of the host. In fact, the “shelter effect” hypothesis predicts that behavioral thermoregulation by intermediate hosts can buffer complex life cycle parasites against temperature extremes, leaving directly transmitted parasites comparatively vulnerable to those extremes (67). The fact that change was observed only among 3+-host parasites suggests an ecological, not physiological, link between climate and parasite abundance.

Although the overall increase in average annual temperatures (1 °C from 1921 to 2019) in Puget Sound over the study period is small compared with marine ecosystems experiencing the fastest rates of warming [3 to 4 °C per century (68)], there is nonetheless ample theoretical and empirical evidence to suggest that temperature could have driven the decline in parasite counts we observed. Importantly, our analysis used annual averages, which disguise more extreme temperature fluctuations present at shorter timescales. For instance, 14 of the 20 warmest months on record occurred since 1990. These fluctuations are potentially highly impactful at the cellular, organismal, and community levels because physiological responses to temperature are highly nonlinear and depend on “thermal safety margins.” These thermal safety margins are considerably smaller in marine ectotherms compared with terrestrial ectotherms (69). Moreover, phenological shifts, often driven in part by degree days, have caused restructuring of food webs and communities in many ecosystems (70), often with as little increase in annual temperature as 1 to 1.5 °C (71, 72). These and other climate change impacts are readily observable today, despite global average warming of 1 °C so far (73).

Parasites are hypothesized to be among the most imperiled species due to their direct sensitivity to environmental change (74) and obligate dependence on hosts that can themselves be sensitive to environmental change (29), but parasite declines and extinctions have been documented only rarely (75). This probably arises from the fact that few data are available to monitor parasite populations, leaving many to decline and disappear unnoticed. We identified 10 parasite taxa that had not been observed in our dataset in the past ≥40 y, including one that had not been observed in 85 y and another that had not been observed in 73 y (*SI Appendix*, Fig. S2). We used consistent detection and identification methods for all hosts in our time series, and all of these parasites had been detected regularly before the dates of their disappearance. None of these parasite taxa were sufficiently abundant to allow us to fit models to individual parasite taxon–level data, and for some, sampling effort was relatively low after the last date of detection (*SI Appendix*, Fig. S2). We highlight them because these taxa may today be so rare that they require more intense search effort to detect, or they may have been extirpated from the Puget Sound ecosystem. With additional research effort, researchers might be able to estimate the likelihood and date of extirpation for these taxa, adding them to the list of parasite species whose losses have been documented (75). It is also worth noting that some parasite taxa (n = 110 taxa, accounting for 443 of the 17,702 parasite individuals detected) were too rare (<5% prevalence) to be included in our analyses; these rare taxa are the ones that are probably the most susceptible to local extirpation, but due to the difficulty of fitting mathematical models to such sparse data, our analysis cannot shed light on their trajectory of change through time.

We had anticipated that statistical tests at the individual parasite taxon level would have low power to detect change over time because parasite data are overdispersed, (76) and there were hard limits set on the amount of replication that we could achieve; that is, only so many appropriate specimens exist, and we were careful to absolutely minimize the amount of sampling we performed for any single host species because our sampling protocol was semidestructive. However, our hierarchical modeling approach allowed us to pool replication across host species and parasite taxa, providing strong statistical power for detecting effects (*SI Appendix*, Table S2). Power was also enhanced by maximizing the length of the time series, which ultimately allowed us to produce one of the longest-term datasets assembled for any group of parasites. We are therefore able to detect temporal trends that would not be detectable in a dataset with a shorter time frame, a single host species, or a single parasite species.

This study was conducted in a single location (Puget Sound, United States), and at the time of this writing, there are no similar data from other locations that can be used to assess the geographic generality of the pattern we report. We measured a decline of 10.9% per decade among parasite taxa with three or more obligately required hosts. For comparison, we can look to taxa of substantial conservation concern and investment, including North American birds (6.3% decline per decade between 1970 and 2017) (12), all terrestrial vertebrates (6.9% decline per decade between 1970 and 2010) (77), and terrestrial insects (8.8% decline per decade between 1925 and 2018) (78). If Puget Sound is representative of many ecosystems and parasite declines are occurring across broad geographic areas at rates similar to those measured here, parasites could match or exceed the rates of decline that have triggered conservation action for other taxa; but this remains to be seen. Parasite conservation is an objective that is gaining increasing support among biologists who study parasites (8). Our work provides empirical evidence for large declines in parasite abundance, suggesting that it may be time to invest in parasite conservation—and that it may even be too late for many parasite taxa.

## Materials and Methods

### Specimen Selection and Collection Site History.

We focused on the metazoan parasites of eight fish species from Puget Sound, Washington, United States, selecting host species that were well represented in natural history collections and that encompassed a broad range of trophic levels, body sizes, habitats, functional feeding groups, and vulnerability to human impacts (*SI Appendix*, Table S1). Specimens were sourced primarily from the University of Washington Fish Collection at the Burke Museum of Natural History and Culture, with additional specimens from other collections to increase temporal scope and resolution (Fig. 1*B* and *SI Appendix*, Table S2). For each specimen examined, we noted locality data from the natural history collection databases and measured the specimen’s TL in centimeters. When the collection location was descriptive but lacked coordinates, we estimated the collection location in decimal degrees using Google Maps.

### Parasitological Dissections.

Each fish was subjected to a comprehensive parasitological dissection (details in refs. 18 and 33). For each parasite identified, we noted its broad taxonomic grouping (Subclass Copepoda, Subclass Hirudinea, Class Monogenea, Class Trematoda, Class Cestoda, Phylum Nematoda, and Class Acanthocephala; *SI Appendix*, Table S3). For flatworms, we stained and mounted specimens before identification (34). For nematodes, we cleared specimens before identification (34). We identified each parasite to the finest possible taxonomic resolution, which in most cases was family or genus level, and classified them into one of two transmission strategies: directly transmitted (i.e., parasites that can be transmitted between conspecific hosts) or complex life cycle (i.e., parasites that are transmitted from one host species to another host species in an obligately required sequence). For complex life cycle parasites, we also estimated the number of obligately required host species based on natural history information (*SI Appendix*, Table S3). Parasites that were identified to species and found in more than one host were recorded under the same parasite taxon name (e.g., *Derogenes varicus*). Larval nematodes of the genera *Anisakis* and *Contracaecum* are known to be host generalists for their fish intermediate/paratenic hosts (35–79); therefore, even though these worms could not be identified to species, we also recorded these under the same parasite taxon name across host species (i.e., *Contracaecum* sp., and *Anisakis* sp.). For all other parasites that were not identified to species, we assumed that individuals found in one host were of a different species than those found in another host and named them accordingly (80, 81) (e.g., *Lepeophtheirus* sp. of Walleye Pollock versus *Lepeophtheirus* sp. of Surf Smelt).

### Potential Environmental Drivers.

In a retrospective study like this one, it is not possible to definitively identify the causal drivers of change in parasite abundance. However, we had access to several long-term environmental datasets and sought to assess the correlation between environmental variables and parasite burden. Our environmental datasets included information on SST (82), heavy metal and organic pollutants (83), and fish host density (36, 37) within Puget Sound.

The temperature and pollutant datasets reflect prevailing conditions in Puget Sound that might have affected all hosts and parasites, while fish density data reflect conditions pertaining primarily to parasites within that fish host. Data on SST were from a continuous record (1921 to 2019) collected at the Race Rocks lighthouse (82); we extracted average monthly SST in degrees Celsius, discarded any year in which more than 1 mo was missing data (n = 4 of 98 y), and obtained an annual average for each year. For each host individual, we matched the year of the host’s collection to the corresponding year from the temperature dataset. Data on pollutants were from a continuous record (1774 to 2005) obtained by coring Puget Sound sediments (83), which yielded values for the concentration of lead, arsenic, zinc, nickel, vanadium, chromium, copper, barium, and beryllium in micrograms per gram of sediment, as well as concentrations of lignin and soil biomarkers, which indicate inputs of terrestrial organic matter. We extracted annual values for each variable from two cores taken near Tacoma and Seattle, WA, in Puget Sound (PS-1 near Tacoma = 47.347167, −122.409667 and PS-4 near Seattle = 47.614967, −122.449017) (83), averaged values within each year across the two cores, and interpolated among years to bridge temporal gaps. Some of the 12 pollutant variables were collinear with one another (*SI Appendix*, Fig. S3), so we performed a principal component analysis to reduce the dimensionality of the pollutant dataset and found that the first two principal components explained 74% of variation (*SI Appendix*, Fig. S4). To account for annual measurement error, we ran a LOESS smoother (locally weighted) on each principal component and then matched the year of each host individual’s collection to the corresponding year from the first and second principal components.

We also had access to data on the density of the fish hosts for six of the eight host species we examined: *Gadus chalcogrammus*, *Hydrolagus colliei, Merluccius productus, Parophrys vetulus* from ref. 36, and *Clupea pallasii* and *Hypomesus pretiosus* from ref. 37. Data from ref. 36 were annual projections of density based on historical data collected from 1946 to 1977, while data from ref. 37 were estimates of catch per unit effort for various Puget Sound basins sampled between 1972 and 2011. For ref. 37, estimates were averaged across basins within each year to obtain an annual estimate of abundance across Puget Sound. For each fish species’ time series, we interpolated among years to bridge temporal gaps and matched the year of each host individual’s collection to the corresponding year from the host density dataset, matching host species to corresponding parasites (i.e., *P. vetulus* density was recorded for *P. vetulus* parasites only).

### Statistical Analyses.

#### Change over time in the abundance of parasites.

The first phase of analysis addressed whether the data provided evidence for change in parasite counts per host individual through time by fitting multilevel generalized linear models with different hierarchical structures and assessing which models were best supported by the data. Details of the model fits and diagnostics are provided in *SI Appendix*, Tables S4–S7, Text S1, and Figs. S2, S5–S7). We chose to use parasite counts per host individual (i.e., parasite abundance) as the response variable because this metric encompasses both prevalence (proportion of hosts infected) and intensity (number of parasite individuals per infected host) (84); it also reflects the population status of parasites (84–86). Briefly, we fit four alternative models, one model without an effect of year on parasite counts and three models with a year effect but with different hierarchical grouping structures: unique effects by parasite taxon with no grouping, unique effects by parasite taxon grouped by the number of obligately required hosts, and unique effects by parasite taxon grouped by broad taxonomic classification (i.e., Subclass Copepoda, Subclass Hirudinea, Class Monogenea, Class Trematoda, Class Cestoda, Phylum Nematoda, and Class Acanthocephala).

The core model predicts the log mean for each observation *I*, based on the host–parasite pair *j*, and parasite species *k.* Because this model has no effect of time, we use this as a point of reference to evaluate evidence for shifting parasite counts through time. The model is as follows:

[1] $$\begin{array}{c} \eta_{i}=\beta_{o\left[j\right]}+X_{lat,i}\beta_{lat\left[k\right]}+X_{len,i}\beta_{len\left[j\right]}+\zeta_{i},\\ y_{i}\sim \mathrm{NegBin}\left(\exp\left(\eta_{i}\right),\phi_{k}\right), \end{array}\;$$

where *β_o_*_[*j*]_ is the intercept for host–parasite pair *j*, *X_lat, i_* is the latitude of observation *i*, *X_len, i_* is the host TL for observation *i*, *β*_*lat*[*k*]_ and *β*_*len*[*j*]_ are the fitted effects of latitude and host TL for taxon *k* and host–parasite pair *j*, respectively, and *ζ*_*i*_ is the random effect of fish specimen identity for observation *i*. We treated all *β* coefficients as random effects, each assumed to be normally distributed with estimated means (*μ_o_*, *μ_lat_*, and *μ_len_*) and SDs (*σ_o_*, *σ_lat_*, and *σ_len_*). The random effects of fish specimen identity were distributed with mean 0 and SD equal to σ_ζ_. We also considered longitude as a potential predictor variable, but the initial model evaluation revealed that longitude had little explanatory power, in part because of the limited longitudinal range of our sample collections (Fig. 1*A*). The parameter *ϕ_k_* is a parasite taxon–specific value of the negative binomial dispersion parameter, which controls the amount of excess variance from the Poisson expectation. These were also treated as random effects, with log(*ϕ*) assumed to be normally distributed around an estimated mean [log(*μ_ϕ_*)] and SD (log *σ_ϕ_*). While this model was designed to account for as many confounding sources of variability in parasite counts as was possible, we note that the data were insufficient to fully account for fine spatiotemporal variation (e.g., localized hotspots in space and time).

To aid model fitting and coefficient interpretation, all continuous predictor variables were scaled to have mean of 0 and SD of 1. For host TL, we scaled sampled fish lengths over observed host–parasite pairs.

We fit three additional models to account for changes in time, all of which include parasite taxon-specific random effects of time on parasite counts, but which differ with respect to whether there is grouping structure by life cycle type (number of obligately required hosts) or broad taxonomic grouping (i.e., Subclass Copepoda, Subclass Hirudinea, Class Monogenea, Class Trematoda, Class Cestoda, Phylum Nematoda, and Class Acanthocephala). The first alternative model asked whether there was an effect of time (year) on parasite counts as follows:

[2] $$\begin{array}{c} \eta_{i}=\beta_{o\left[j\right]}+X_{lat,i}\beta_{lat\left[k\right]}+X_{len,i}\beta_{len\left[j\right]}+\zeta_{i}+U_{i}\gamma_{\left[k\right]},\\ \gamma_{\left[k\right]}\sim N\left(\mu_{\gamma },\sigma_{\gamma }\right), \end{array}$$

where *U_i_* is the scaled year for observation *i*, *γ*_[*k*]_ is the random effect of time for taxa *k*, *μ*_*γ*_ is the mean of the random effects across all taxa, and *σ*_*γ*_ the SD of the time effects.

The remaining two models asked whether the mean of the time effects (*γ*_[__*k*__]_) differed depending on the number of obligately required hosts or broad taxonomic grouping as follows:

[3] $$\begin{array}{c} \gamma_{\left[k\right]}\sim N\left(\mu_{\gamma \left[l\right]},\sigma_{\gamma }\right)\;\mathrm{group}\;\mathrm{by}\;\mathrm{no}.\;\mathrm{of}\;\mathrm{hosts},\\ \gamma_{\left[k\right]}\sim N\left(\mu_{\gamma \left[p\right]},\sigma_{\gamma }\right)\;\mathrm{group}\;\mathrm{by}\;\mathrm{taxonomic}\;\mathrm{group}, \end{array}$$

where *μ*_*γ*[*l*]_ and *μ*_*γ*[*p*]_ are distinct means for groups based on the number of obligately required hosts or broad taxonomic classification, respectively. These additions required modifications to the intercept term as follows:

[4] $$\begin{array}{c} \beta_{o,i}=\beta_{o,\left[j\right]}+\beta_{o,\left[g\right]},\\ \beta_{o,\left[j\right]}\sim N\left(0,\;\sigma_{\beta_{o}}^{2}\right),\\ \beta_{o,\left[g\right]}\sim N\left(\mu_{\beta_{o}},\;\sigma_{\beta_{o,g}}\right), \end{array}$$

where *β*_*o*[_*_g_*_]_ is the mean intercept for species in classification *g* (either the number of obligately required hosts or broad taxonomic classification). A description of all fixed effects parameters is listed in *SI Appendix*, Table S7.

We judged the degree of support for each model using small sample size–corrected AIC (AICc) calculated from the number of fixed effects parameters.

#### Assessing environmental correlates of change over time in the abundance of parasites.

The second phase of analysis aimed to link temporal changes in parasite abundance to putative drivers of change. Here, we focused only on those taxa with at least three obligately required host species because phase 1 results indicated that these taxa showed the strongest change through time. Our aim was to ask whether changes predicted by a time effect (i.e., some latent time-dependent process) were comparable with those predicted by measurable environmental effects, namely SST, pollutant levels, or host density.

Ideally, we would use model selection to ask which alternative combination of effects best explains the observed declines in parasite counts. However, the time span of these alternative drivers did not overlap extensively (Fig. 1*B*), so truncating the data to include only those years with data availability across all environmental variables led to omission of large parts of the data, including years when there were relatively rapid changes. For that reason, we fit models to distinct subsets of the data spanning the range of data availability for each environmental variable and compared estimated effect sizes to evaluate which factors might have contributed most strongly to the observed change through time.

We used the model described in Eq. **2**, but substituted scaled SST, scaled pollutant PCA 1 or 2, or host density for year (i.e., *U_i_* could represent any of these numerical predictor variables). When needed, we introduced multiple vectors *U*_*p*_ to account for multiple drivers as follows:

[5] $$\eta_{i}=\beta_{o,\left[j\right]}+X_{lat,i}\beta_{lat\left[k\right]}+X_{len,i}\beta_{len\left[j\right]}+\zeta_{i}+\sum_{p=1}^{n_{p}}U_{i,p\gamma \left[k\right],p},$$

where *n_p_* is the number of different time-varying predictor variables, and *γ*_[*k*],*p*_ is the effect of predictor variable *p* for species *k*.

Model diagnostics were performed on all models supported by the data. These included Q–Q plots, standardized residuals versus predicted values plots, and several statistical tests of goodness of fit including the Kolmogorov–Smirnov D statistic and a simulation-based test for overdispersion (87).

All models were fit using Template Model Builder v 1.7.22 (88) in R v 4.1.2 (89).

## Supplementary Material

Appendix 01 (PDF)

## Data Availability

The data that support the findings of this study are openly available in the Dryad Digital Repository: https://doi.org/10.5061/dryad.fqz612jwf. The code used to produce the statistical results reported herein is openly available via GitHub at https://github.com/wood-lab/Wood_et_al_2022_PNAS.  1) Parasite abundance data, host meta-data, data on environmental correlates of parasite abundance change.
