# Supplementary material for: A reconstruction of parasite burden reveals one century of climate-associated parasite decline
Source: Proc Natl Acad Sci U S A. 2023 Jan 9;120(3):e2211903120. doi: 10.1073/pnas.2211903120 (PMC9934024; doi:10.1073/pnas.2211903120)
Supplement: Supplementary file 1 — Appendix 01 (PDF) [file pnas.2211903120.sapp.pdf]

Supplementary Information Table S1. Ecological attributes of the eight host species selected for study. Data from FishBase (Froese et al. 2022). Habitat indicates whether the species is primarily neritic (i.e., in the water column) or demersal (i.e., near or on the benthos). Vulnerability score given out of 100 points, with higher scores indicating higher vulnerability to human impacts (Cheung et al. 2005).

Host Latin name	Host common name	Order	Trophic level	Habitat	Schooling	Vulnerability
Clupea pallasii	Pacific Herring	Clupeiformes	3.2	Neritic	Yes	Low to moderate (28)
Embiotoca lateralis	Striped Sea Perch	Ovalentaria	3.3	Demersal	No	Moderate (39)
Hydrolagus colliei	Spotted Ratfish	Chimaeriformes	3.7	Demersal	No	Moderate to high (50)
Hypomesus pretiosus	Surf Smelt	Osmeriformes	3.4	Demersal	During spawning	Low to moderate (31)
Merluccius productus	Pacific Hake	Gadiformes	4.4	Neritic	Yes	High (60)
Parophrys vetulus	English Sole	Pleuronectiformes	3.4	Demersal	No	Moderate (43)
Sebastes caurinus	Copper Rockfish	Perciformes	4.1	Demersal	No	High (58)
Gadus chalcogrammus	Walleye Pollock	Gadiformes	3.6	Demersal	During spawning	Moderate to high (45)

Supplementary Information Table S2. Number of individuals of each species of fish dissected in each decade between 1880 and 2019. Cells are conditionally formatted such that more intense color indicates greater replication.

**Fish species**	**1880**	**1890**	**1900**	**1910**	**1920**	**1930**	**1940**	**1950**	**1960**	**1970**	**1980**	**1990**	**2000**	**2010**
Clupea pallasii (Pacific herring)	0	0	10	0	14	14	5	2	14	10	14	14	0	10
Embiotoca lateralis (Striped Sea Perch)	0	0	5	0	3	14	14	14	17	12	10	5	7	4
Hydrolagus colliei (Spotted Ratfish)	0	0	0	0	0	1	5	8	12	5	6	6	1	2
Hypomesus pretiosus (Surf Smelt)	1	0	2	0	20	13	16	0	15	5	4	0	0	3
Merluccius productus (Pacific Hake)	0	0	0	0	0	14	3	1	9	2	14	14	0	11
Parophrys vetulus (English sole)	0	0	0	0	0	14	14	6	8	11	13	13	16	14
Sebastes caurinus (Copper Rockfish)	0	2	0	0	14	14	14	14	14	7	3	0	5	0
Gadus chalcogrammus (Walleye Pollock)	0	0	4	0	2	14	14	5	3	12	4	14	12	14

Supplementary Information Table S3. Natural history information for each of 85 parasite taxa that occurred at >5% in a single host species. We identified each parasite to the lowest possible taxonomic level, noting its broad taxonomic grouping (Subclass Copepoda, Subclass Hirudinea, Class Monogenea, Class Trematoda, Class Cestoda, Phylum Nematoda, Phylum Acanthocephala), life stage (larval, adult, or undetermined), and transmission strategy (complex life cycle versus directly transmitted). Number of hosts indicates the number of obligately required host species in the life cycle (= 1 for directly transmitted parasites, >1 for complex life cycle parasites). See Figure 2 in the main text for information on how each parasite taxon changed in abundance over time.

**Parasite code**	**Host species**	**Parasite taxonomic identification**	**Broad taxonomic grouping**	**Life stage**	**Transmission strategy**	**Number of hosts**
COP-CP-BOM	Pacific Herring (*Clupea pallasii*)	*Bomolochus bellones*	Copepoda	Adult	Directly transmitted	1
COP-CP-SP1	Pacific Herring (*Clupea pallasii*)	Copepod sp.	Copepoda	Adult	Directly transmitted	1
COP-EL-BOM	Striped Sea Perch (*Embiotoca lateralis*)	*Bomolochus cuneatus*	Copepoda	Adult	Directly transmitted	1
COP-EL-CLA	Striped Sea Perch (*Embiotoca lateralis*)	*Clavella* sp.	Copepoda	Adult	Directly transmitted	1
COP-GC-LEA	Walleye Pollock (*Gadus chalcogrammus*)	*Lepeophtheirus* sp.	Copepoda	Adult	Directly transmitted	1
COP-GC-LEL	Walleye Pollock (*Gadus chalcogrammus*)	*Lepeophtheirus* sp.	Copepoda	Larval	Directly transmitted	1
COP-GC-SP1	Walleye Pollock (*Gadus chalcogrammus*)	Copepod sp.	Copepoda	Undetermined	Directly transmitted	1
COP-HP-LEP	Surf Smelt (*Hypomesus pretiosus*)	*Lepeophtheirus* sp.	Copepoda	Adult	Directly transmitted	1
COP-MP-PAR	Pacific Hake (*Merluccius productus*)	*Parabrachiella* sp.	Copepoda	Adult	Directly transmitted	1

COP-MP-SP1	Pacific Hake (*Merluccius productus*)	Copepod sp.	Copepoda	Adult	Directly transmitted	1
COP-PV-LEP	English Sole (*Parophrys vetulus*)	*Lepeophtheirus* sp.	Copepoda	Larval	Directly transmitted	1
COP-SC-CLA	Copper Rockfish (*Sebastes caurinus*)	*Clavella* sp.	Copepoda	Adult	Directly transmitted	1
COP-SC-LER	Copper Rockfish (*Sebastes caurinus*)	Lernaeopodid sp.	Copepoda	Adult	Directly transmitted	1
COP-SC-SP1	Copper Rockfish (*Sebastes caurinus*)	Copepod sp.	Copepoda	Adult	Directly transmitted	1
HIR-PV-OCE	English Sole (*Parophrys vetulus*)	*Oceanobdella pallida*	Hirudinea	Adult	Directly transmitted	1
MON-CP-GYR	Pacific Herring (*Clupea pallasii*)	*Gyrodactylus* sp.	Monogenea	Adult	Directly transmitted	1
MON-HC-CHI	Spotted Ratfish (*Hydrolagus colliei*)	*Chimaericola* sp.	Monogenea	Adult	Directly transmitted	1
MON-MP-ANT	Pacific Hake (*Merluccius productus*)	*Anthocotyle merlucci*	Monogenean	Adult	Directly transmitted	1
MON-SC-MI1	Copper Rockfish (*Sebastes caurinus*)	*Microcotyle* sp. 1	Monogenea	Adult	Directly transmitted	1
MON-SC-MI2	Copper Rockfish (*Sebastes caurinus*)	*Microcotyle* sp. 2	Monogenea	Adult	Directly transmitted	1
TRE-CP-HEM	Pacific Herring (*Clupea pallasii*)	Hemiurid sp.	Trematoda	Adult	Complex life cycle	3
TRE-CP-LEC	Pacific Herring (*Clupea pallasii*)	*Lecithaster* sp.	Trematoda	Adult	Complex life cycle	3
TRE-CP-ME1	Pacific Herring (*Clupea pallasii*)	Metacercaria sp. 1	Trematoda	Larval	Complex life	3

					cycle	
TRE-CP-ME2	Pacific Herring (*Clupea pallasii*)	Metacercaria sp. 2	Trematoda	Larval	Complex life cycle	3
TRE-CP-PRO	Pacific Herring (*Clupea pallasii*)	*Pronoprymna* sp.	Trematoda	Adult	Complex life cycle	3
TRE-CP-SP1	Pacific Herring (*Clupea pallasii*)	Trematode sp.	Trematoda	Adult	Complex life cycle	3
TRE-EL-MET	Striped Sea Perch (*Embiotoca lateralis*)	Metacercaria sp.	Trematoda	Larval	Complex life cycle	3
TRE-GC-HEM	Walleye Pollock (*Gadus chalcogrammus*)	Hemiurid sp.	Trematoda	Adult	Complex life cycle	3
TRE-GC-LEC	Walleye Pollock (*Gadus chalcogrammus*)	*Lecithaster* sp.	Trematoda	Adult	Complex life cycle	3
TRE-GC-LEP	Walleye Pollock (*Gadus chalcogrammus*)	*Lepidapedon* sp.	Trematoda	Adult	Complex life cycle	3
TRE-GC-ME1	Walleye Pollock (*Gadus chalcogrammus*)	Metacercaria sp. 1	Trematoda	Larval	Complex life cycle	3
TRE-GC-ME2	Walleye Pollock (*Gadus chalcogrammus*)	Metacercaria sp. 2	Trematoda	Larval	Complex life cycle	3
TRE-GC-POD	Walleye Pollock (*Gadus chalcogrammus*)	*Podocotyle* sp.	Trematoda	Adult	Complex life cycle	3
TRE-GC-SP1	Walleye Pollock (*Gadus chalcogrammus*)	Trematode sp.	Trematoda	Adult	Complex life cycle	3
TRE-HC-GON	Spotted Ratfish (*Hydrolagus colliei*)	*Gonocerca* sp.	Trematoda	Adult	Complex life cycle	3
TRE-HP-LEC	Surf Smelt (*Hypomesus pretiosus*)	*Lecithaster* sp.	Trematoda	Adult	Complex life cycle	3

TRE-HP-MET	Surf Smelt (*Hypomesus pretiosus*)	Metacercaria sp.	Trematoda	Larval	Complex life cycle	3
TRE-MP-APO	Pacific Hake (*Merluccius productus*)	*Aporocotyle margolisi*	Trematoda	Adult	Complex life cycle	2
TRE-MP-LEC	Pacific Hake (*Merluccius productus*)	*Lecithaster* sp.	Trematoda	Adult	Complex life cycle	3
TRE-MP-MET	Pacific Hake (*Merluccius productus*)	Trematode metacercaria	Trematoda	Larval	Complex life cycle	3
TRE-MP-POD	Pacific Hake (*Merluccius productus*)	*Podocotyle* sp.	Trematoda	Adult	Complex life cycle	3
TRE-MP-SP1	Pacific Hake (*Merluccius productus*)	Trematode sp.	Trematoda	Adult	Complex life cycle	3
TRE-PV-ME1	English Sole (*Parophrys vetulus*)	Trematode metacercaria sp. 1	Trematoda	Larval	Complex life cycle	3
TRE-PV-ME2	English Sole (*Parophrys vetulus*)	Trematode metacercaria sp. 2	Trematoda	Larval	Complex life cycle	3
TRE-PV-SP1	English Sole (*Parophrys vetulus*)	Trematode sp. 1	Trematoda	Adult	Complex life cycle	3
TRE-PV-SP2	English Sole (*Parophrys vetulus*)	Trematode sp. 2	Trematoda	Adult	Complex life cycle	3
TRE-SC-LEC	Copper Rockfish (*Sebastes caurinus*)	*Lecithochirium* sp.	Trematoda	Adult	Complex life cycle	3
TRE-SC-MET	Copper Rockfish (*Sebastes caurinus*)	Trematode metacercaria	Trematoda	Larval	Complex life cycle	3
TRE-SC-OPE	Copper Rockfish (*Sebastes caurinus*)	*Opechona* sp.	Trematoda	Adult	Complex life cycle	3
TRE-SC-SP1	Copper Rockfish (*Sebastes	Trematode sp. 1	Trematoda	Adult	Complex life	3

	caurinus)				cycle	
TRE-SC-SP2	Copper Rockfish (*Sebastes caurinus*)	Trematode sp. 2	Trematoda	Adult	Complex life cycle	3
TRE-SE-DER	Copper Rockfish (*Sebastes caurinus*), Pacific Hake (*Merluccius productus*), Walleye Pollock (*Gadus chalcogrammus*)	*Derogenes varicus*	Trematoda	Adult	Complex life cycle	3
TRE-SE-PRO	Pacific Herring (*Clupea pallasii*), Surf Smelt (*Hypomesus pretiosus*)	*Pronoprymna petrowi*	Trematoda	Adult	Complex life cycle	3
CES-CP-SP1	Pacific Herring (*Clupea pallasii*)	Cestode sp.	Cestoda	Larval	Complex life cycle	3
CES-CP-SP2	Pacific Herring (*Clupea pallasii*)	Cestode sp.	Cestoda	Larval	Complex life cycle	3
CES-GC-SP1	Walleye Pollock (*Gadus chalcogrammus*)	Cestode sp.	Cestoda	Undetermined	Complex life cycle	3
CES-GC-SP2	Walleye Pollock (*Gadus chalcogrammus*)	Cestode sp.	Cestoda	Larval	Complex life cycle	3
CES-GC-SP3	Walleye Pollock (*Gadus chalcogrammus*)	Cestode sp.	Cestoda	Larval	Complex life cycle	3
CES-HC-GYR	Spotted Ratfish (*Hydrolagus colliei*)	*Gyrocotyle* sp.	Cestoda	Adult	Complex life cycle	2
CES-HP-SP1	Surf Smelt (*Hypomesus pretiosus*)	Cestode sp. 1	Cestoda	Larval	Complex life cycle	3
CES-MP-CLE	Pacific Hake (*Merluccius productus*)	*Clestobothrium crassiceps*	Cestoda	Adult	Complex life cycle	2
CES-MP-NYB	Pacific Hake (*Merluccius productus*)	*Nybelinia surmenicola*	Cestoda	Larval	Complex life cycle	4

CES-MP-SP1	Pacific Hake (*Merluccius productus*)	Cestode sp. 1	Cestoda	Larval	Complex life cycle	3
CES-MP-SP2	Pacific Hake (*Merluccius productus*)	Cestode sp. 2	Cestoda	Larval	Complex life cycle	3
CES-MP-SP3	Pacific Hake (*Merluccius productus*)	Cestode sp. 3	Cestoda	Larval	Complex life cycle	3
NEM-CP-SP1	Pacific Herring (*Clupea pallasii*)	Nematode sp.	Nematoda	Both larval and adult	Complex life cycle	2
NEM-EL-CUC	Striped Sea Perch (*Embiotoca lateralis*)	*Cucullanellus kanabus*	Nematoda	Adult	Complex life cycle	2
NEM-GC-ASC	Walleye Pollock (*Gadus chalcogrammus*)	*Ascarophis* sp.	Nematoda	Adult	Complex life cycle	2
NEM-GC-SP1	Walleye Pollock (*Gadus chalcogrammus*)	Nematode sp.	Nematoda	Both larval and adult	Complex life cycle	2
NEM-HP-SP1	Surf Smelt (*Hypomesus pretiosus*)	Nematode sp.	Nematoda	Both larval and adult	Complex life cycle	2
NEM-MP-ASC	Pacific Hake (*Merluccius productus*)	*Ascarophis* sp.	Nematoda	Adult	Complex life cycle	2
NEM-MP-SP1	Pacific Hake (*Merluccius productus*)	Nematode sp.	Nematoda	Both larval and adult	Complex life cycle	2
NEM-PV-CAP	English Sole (*Parophrys vetulus*)	*Capillaria parophrysi*	Nematoda	Adult	Complex life cycle	2
NEM-PV-CLA	English Sole (*Parophrys vetulus*)	*Clavinema mariae*	Nematoda	Adult	Complex life cycle	2
NEM-PV-CUC	English Sole (*Parophrys vetulus*)	*Cucullanus annulatus*	Nematoda	Adult	Complex life cycle	2
NEM-PV-SP1	English Sole (*Parophrys vetulus*)	Nematode sp.	Nematoda	Both larval	Complex life	2

				and adult	cycle	
NEM-PV-SPR	English Sole (*Parophrys vetulus*)	Spirurid sp.	Nematoda	Adult	Complex life cycle	2
NEM-SC-ASC	Copper Rockfish (*Sebastes caurinus*)	*Ascarophis sebastodis*	Nematoda	Adult	Complex life cycle	2
NEM-SE-ANI	Pacific Hake (*Merluccius productus*), Walleye Pollock (*Gadus chalcogrammus*)	*Anisakis* sp.	Nematoda	Larval	Complex life cycle	3
NEM-SE-CON	Copper Rockfish (*Sebastes caurinus*), English Sole (*Parophrys vetulus*), Pacific Hake (*Merluccius productus*), Pacific Herring (*Clupea pallasii*), Spotted Ratfish (*Hydrolagus colliei*), Striped Sea Perch (*Embiotoca lateralis*), Surf Smelt (*Hypomesus pretiosus*), Walleye Pollock (*Gadus chalcogrammus*)	*Contracaecum* sp.	Nematoda	Larval	Complex life cycle	3
ACA-CP-COR	Pacific Herring (*Clupea pallasii*)	*Corynosoma* sp.	Acanthocephala	Larval	Complex life cycle	2
ACA-HP-ECH	Surf Smelt (*Hypomesus pretiosus*)	*Echinorhynchus gadi*	Acanthocephala	Larval	Complex life cycle	2
ACA-PV-SP1	English Sole (*Parophrys vetulus*)	Acanthocephalan sp.	Acanthocephala	Undetermi ned	Complex life cycle	2
ACA-SC-COR	Copper Rockfish (*Sebastes caurinus*)	*Corynosoma* sp.	Acanthocephala	Larval	Complex life cycle	2
ACA-SC-ECH	Copper Rockfish (*Sebastes caurinus*)	*Echinorhynchus* sp.	Acanthocephala	Larval	Complex life cycle	2

Supplementary Information Table S4. Model selection via AIC for the first phase of analysis. All models contain the null group of random effects (parasite taxa, host-parasite pairing, host length, latitude) as described in Eq. 1. Parasite taxon indicates the parasite taxonomic identification (see column in **Supplementary Information Table S3**). Broad parasite taxonomic grouping indicates the parasite's membership in the following groups: Subclass Copepoda, Subclass Hirudinea, Class Monogenea, Class Trematoda, Class Cestoda, Phylum Nematoda, Phylum Acanthocephala (see column in **Supplementary Information Table S3**).

Model #	Additional random effects	# fixed effect parameters	-Log(likelihood)	ΔAIC
1	None	9	8558.8	62.45
2	Year by parasite taxon	11	8525.6	1.12
3	Year by parasite taxon and number of required hosts	14	8522.0	0
4	Year by broad parasite taxonomic grouping	18	8519.7	3.32

Supplementary Information Table S5. Model selection results relating putative environmental drivers to changes in parasite counts for parasite taxa with three or more obligately required hosts. Covariates are the estimated mean effect size (on scaled predictor variables) for each model. Models with contaminants have two covariate variables, so ordination axis 1 corresponds to first covariate and ordination axis 2 corresponds to second covariate. Model comparisons are only valid within sub-groups defined by the leftmost column.

		Environmental Covariate (SE)		Year (SE)
Predictor and models	ΔAIC	1st covariate	2nd covariate	
Annual temperature				
Null	30.1	-	-	-
Temperature	9.8	-0.33 (0.09)	-	-
Year	1.1	-	-	-0.31 (0.11)
Temperature + Year	0	-0.2 (0.1)	-	-0.18 (0.12)
Contaminants				
Null	43.6	-	-	-
Contaminants	18.2	-0.01 (0.11)	-0.12 (0.06)	-
Year	12.8	-	-	-0.19 (0.12)
Contaminants + Year	0	-0.04 (0.11)	-0.16 (0.1)	-0.11 (0.07)
Host density				
Null	0	-	-	-
Host density	1.4	-0.02 (0.19)	-	-
Year	4.1	-	-	-0.01 (0.19)
Host density + year	5.5	-0.01 (0.2)		-0.05 (0.19)

Supplementary Information Table S6a. Estimated fixed effects parameters for model 1 (no year effect) fit in phase 1 of the analysis.

Parameter	Maximum likelihood estimate	SE
$\mu_{\beta o}$	-0.981	0.165
$\sigma_{\beta o}$	1.524	0.120
$\mu_{\beta lat}$	-0.116	0.078
$\sigma_{\beta lat}$	0.485	0.079
$\mu_{\beta len}$	0.481	0.083
$\sigma_{\beta len}$	0.648	0.072
σ_{ζ}	0.503	0.049
$\log(\mu_{\phi})$	-1.754	0.113
$\log(\sigma_{\phi})$	0.853	0.089

Supplementary Information Table S6b. Estimated fixed effects parameters fit to model 2 (year effect by parasite taxon) in phase 1 of the analysis.

Parameter	Maximum likelihood estimate	SE
μ_{γ}	-0.190	0.083
σ_{γ}	0.565	0.085
$\mu_{\beta o}$	-1.088	0.165
$\sigma_{\beta o}$	1.504	0.119
$\mu_{\beta lat}$	-0.125	0.073
$\sigma_{\beta lat}$	0.428	0.076
$\mu_{\beta len}$	0.510	0.081
$\sigma_{\beta len}$	0.632	0.071
σ_{ζ}	0.457	0.050
$\log(\mu_{\phi})$	-1.678	0.112
$\log(\sigma_{\phi})$	0.833	0.090

Supplementary Information Table S6c. Estimated fixed effects parameters fit to model 3 (year effect by parasite taxon and number of obligately required hosts in life cycle) in phase 1 of the analysis.

Parameter	Maximum likelihood estimate	SE
$\mu_{\gamma,l=1}$	-0.030	0.160

$\mu_{\gamma,l=2}$	0.010	0.160
$\mu_{\gamma,l=3+}$	-0.350	0.110
σ_{γ}	0.550	0.080
$\mu_{\beta o}$	-1.230	0.290
$\sigma_{\beta o}$	1.450	0.120
$\sigma_{\beta o[g=l]}$	0.400	0.250
$\mu_{\beta,lat}$	-0.120	0.070
$\sigma_{\beta,lat}$	0.430	0.080
$\mu_{\beta,len}$	0.510	0.080
$\sigma_{\beta l,en}$	0.630	0.070
σ_{ζ}	0.460	0.050
$\log(\mu_{\phi})$	-1.680	0.110
$\log(\sigma_{\phi})$	0.830	0.090

Supplementary Information Table S6d. Estimated fixed effects parameters fit to model 4 (year effect by parasite taxon and by broader taxonomic grouping).

Parameter	Maximum likelihood estimate	SE
μ_{γ},annelid	-0.180	0.350
μ_{γ},acanthocephalan	-0.430	0.220
μ_{γ}, cestode	-0.030	0.190
μ_{γ},copepod	0.670	0.740
μ_{γ},monogenean	-0.230	0.360
μ_{γ}, nematode	-0.060	0.180
μ_{γ}, trematode	-0.260	0.130
σ_{γ}	0.560	0.090
$\mu_{\beta o}$	-1.350	0.300
$\sigma_{\beta o}$	1.380	0.110
$\sigma_{\beta o[g=p]}$	0.570	0.230
$\mu_{\beta lat}$	-0.120	0.070
$\sigma_{\beta lat}$	0.430	0.080
$\mu_{\beta len}$	0.510	0.080
$\sigma_{\beta len}$	0.620	0.070
σ_{ζ}	0.460	0.050
$\log(\mu_{\phi})$	-1.680	0.110
$\log(\sigma_{\phi})$	0.840	0.090

Supplementary Information Table S7. Fixed effects parameters and descriptions. See Equations 1–4 in main text.

Parameter	***Description***
$\mu_{\beta o}$	mean of intercepts over *j* host parasite pairs (model 1 & 2) or *g* groupings (model 3 & 4)
$\sigma_{\beta o}$	standard deviation of intercepts over *j* host parasite pairs (model 1 & 2) or *g* groupings (model 3 & 4)
$\mu_{\beta,lat}$	mean effect of latitude over *k* parasite taxa
$\sigma_{\beta,lat}$	standard deviation of the effect of latitude over *k* parasite taxa
$\mu_{\beta,len}$	Mean effect of host length over *j* host- parasite pairs
$\sigma_{\beta,len}$	standard deviation of the effect of host length over *j* host- parasite pairs
σ_{ζ}	standard deviation of the effect of individual fish specimen
$\log(\mu_{\phi})$	log (mean) of the dispersion parameter of the negative binomial distribution over *k* parasite species
$\log(\sigma_{\phi})$	log (standard deviation) of the dispersion parameter of the negative binomial distribution over *k* parasite species
μ_{γ}	mean effect of year over *k* parasite taxa
σ_{γ}	standard deviation of year effect over parasite taxa
$\mu_{\gamma,l}$	mean effect of year for parasite taxa with *l* hosts
$\mu_{\gamma,p}$	mean effect of year for parasite taxa in taxonomic grouping *p*
$\sigma_{\beta o[g=l]l}$	standard deviation of intercepts over *l* host groupings
$\sigma_{\beta o[g=p]}$	standard deviation of intercepts over *p* taxonomic groupings
μ_{z}	mean effect of covariate *z* over parasite taxa
σ_{z}	standard deviation of effect of covariate *z* over parasite taxa

Supplementary Information Text S1. ***Model Diagnostics*** **–** We performed typical diagnostic analyses to confirm goodness of fit, identify residual patterns, and to evaluate overdispersion. To this end, we used a simulation approach, whereby we simulated data from the fitted model conditional on the fitted random effects. We modified functions in the DHARMa R package (Hartig 2020) to perform calculations on our TMB model output. The best-fitting model from phase 1 (using all data, fitting the year effect by number of obligately required hosts in the life cycle) indicated no diagnostic issues (**Supplementary Information Figure S5**). The quantile plot indicated no systematic deviation away from the assumed (negative binomial) distribution (Kolmogrov-Smirnoff D statistic = 0.0106, p-value = 0.29). To test for overdispersion, we compared the observed variance of the residuals to simulated variance of the residuals, as implemented in the DHARMa package. We found modest evidence of overdispersion of the data from the fitted model (p = 0.112).

Diagnostic plots from the second phase of analysis also did not reveal any serious model fit issues (**Supplementary Information Figure S6**). The observed residuals were not perfectly aligned with the expectations (Kolmogrov-Smirnov D = 0.027, p <0.001). However, the magnitude of the deviation was relatively small. The simulation test for under- or over-dispersion (two-sided) was not significant (p = 0.712). There was some evidence of patterning in the scaled residuals (**Supplemental Information Figure S6**), particularly at large predicted values where the scaled residuals tended to increase above the expected value of 0.5. Outliers were detected at the upper range of the residuals.

Supplementary Information Figure S1. Occurrence (i.e., presence = 1, absence = 0) of 10 parasite taxa that were not observed in the past ≥40 years. Coordinates are jittered in the y-direction to allow visualization of overlapping points. The last year of observation for each parasite taxon is noted with a vertical dashed line. Parasite codes given on the y-axis correspond to **Supplementary Table S3**. Directly transmitted parasites (i.e., parasites with only one obligately required host species) are indicated in yellow, complex life cycle parasites with three obligately required host species are indicated in light blue, and complex life cycle parasites with four obligately required host species are indicated in dark blue. Although we do not have enough observations to statistically analyze the likelihood of true absence for these taxa, they may be extirpated from the system; only more intensive sampling effort will reveal whether this is the case. Total number of host individuals dissected for each parasite taxon (before / after year of last detection): COP-HP-LEP = 79 (32 / 47), CES-HP-SP1 = 79 (50 / 29), TRE-SC-OPE = 87 (70 / 17), TRE-HC-GON = 46 (20 / 26), TRE-HP-MET = 79 (72 / 7), CES-MP-NYB = 68 (29 / 39), TRE-SC-SP2 = 87 (74 / 13), TRE-PV-SP1 = 109 (48 / 61), TRE-SC-MET = 87 (79 / 8), TRE-SC-SP1 = 87 (79 / 8).

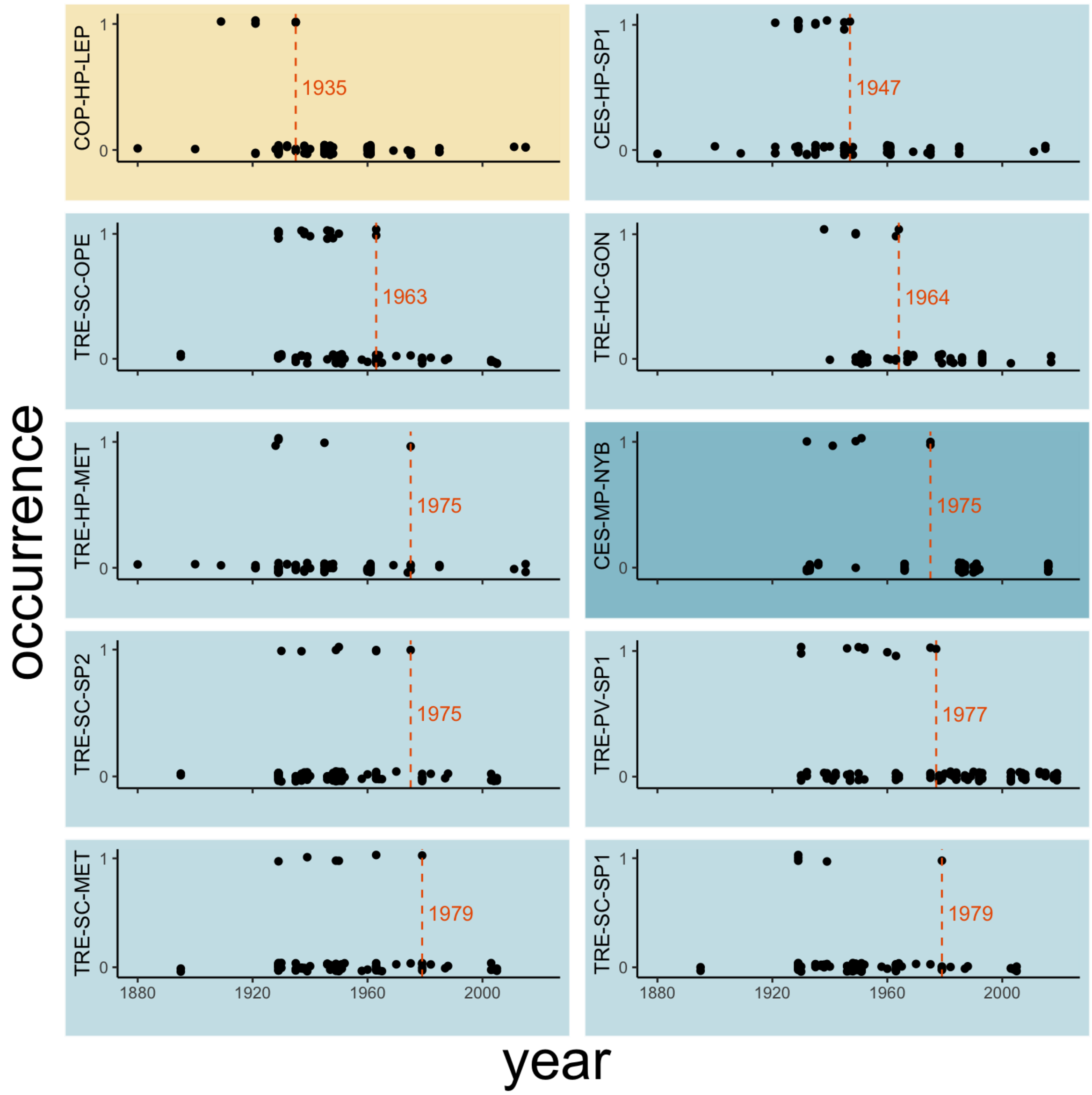

Supplementary Information Figure S2. Estimated effect size of sediment contaminant levels (showing ordination axis 1) and year for each taxa. Each point is a maximum likelihood estimate and lines denote standard errors.

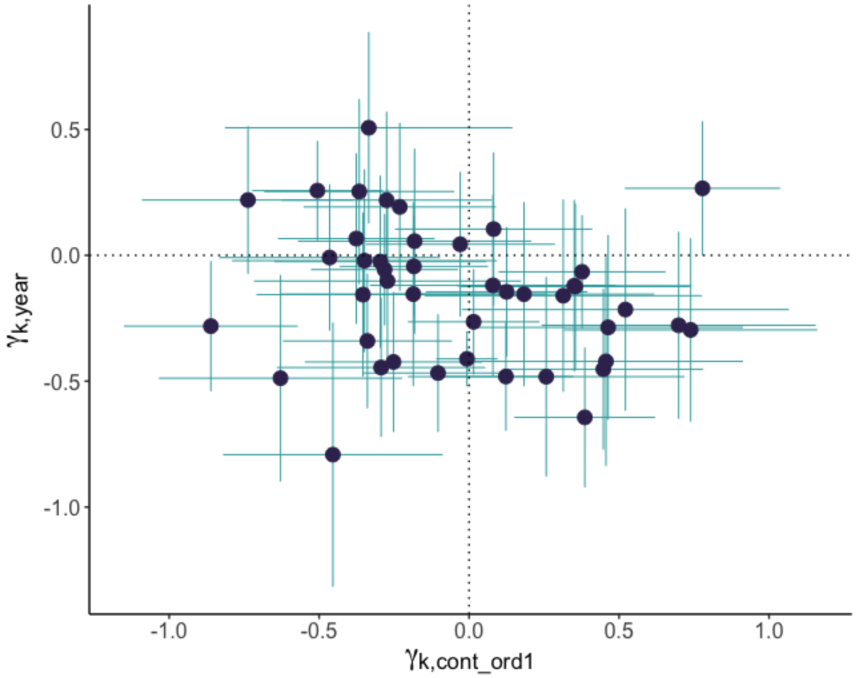

Supplementary Information Figure S3. Correlation matrix displaying the correlation coefficient for all pairs of variables among the 12 pollutant variables extracted from Brandenberger et al. (2008) and year. Redder colors indicate more negative correlations and bluer colors indicate more positive correlations. Pb = lead, As = arsenic, Zn = zinc, Ni = nickel, V = vanadium, Cr = chromium, Cu = copper, Ba = barium, Be = beryllium (all in micrograms per gram of sediment), Sig8_lignin = sum total of the eight major lignin-derived phenols per gram of sediment in mg/g, Lamb8 = carbon-normalized yields of the eight major lignin-derived phenols in mg per 100 mg of organic carbon, and Bd.V_soil_biomarker = ratio of 3,5-dihydroxybenzoic acid (3,5Bd) to total vanillyl phenols (3,5Bd/V), a tracer for soil organic matter inputs to aquatic ecosystems. Note that there are some strong correlations between variables, which is why we chose to reduce the dimensionality of this dataset using principal component analysis.

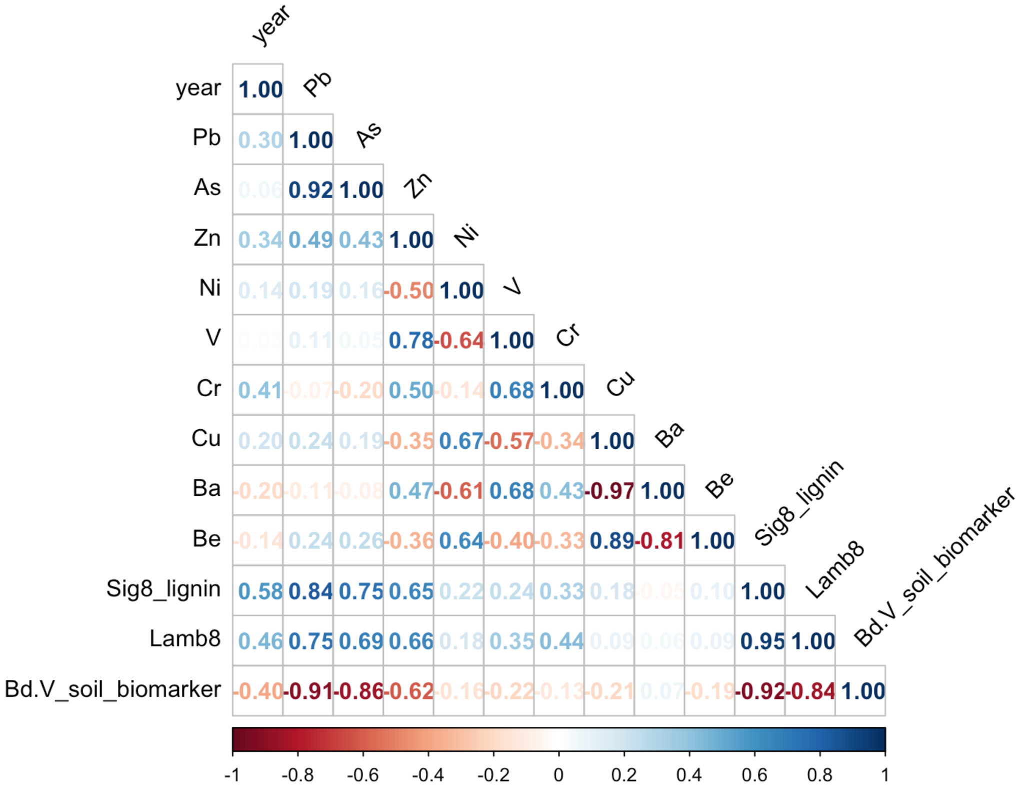

Supplementary Information Figure S4. Graphical depiction of the first two principal components axis of sediment contaminant data. Each point is a yearly observation, and the red lines depict the loadings of each contaminant on each of the first two axes. The numbers in parenthesis on axis titles depict the proportion of variance explained by each principal component axis.

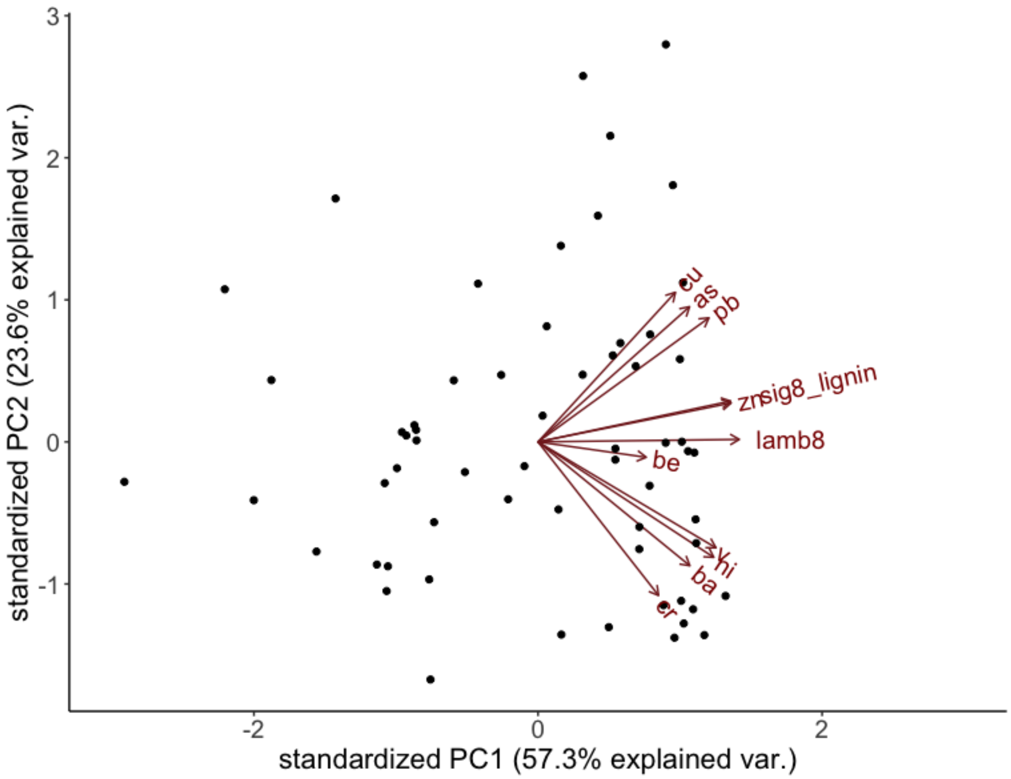

Supplementary Information Figure S5. Precision-weighted histograms of random effects of time by number of obligately required hosts in life cycle (1, 2, 3+), for models with no grouping (top row) and grouping by number of hosts (bottom row). Evidence for shrinkage from partial pooling is particularly evident for taxa with 1 or 3+ obligate hosts. In comparison, taxa with 2 obligate hosts exhibited a wide range of effect sizes, and this was not reduced by modeling the grouping structure.

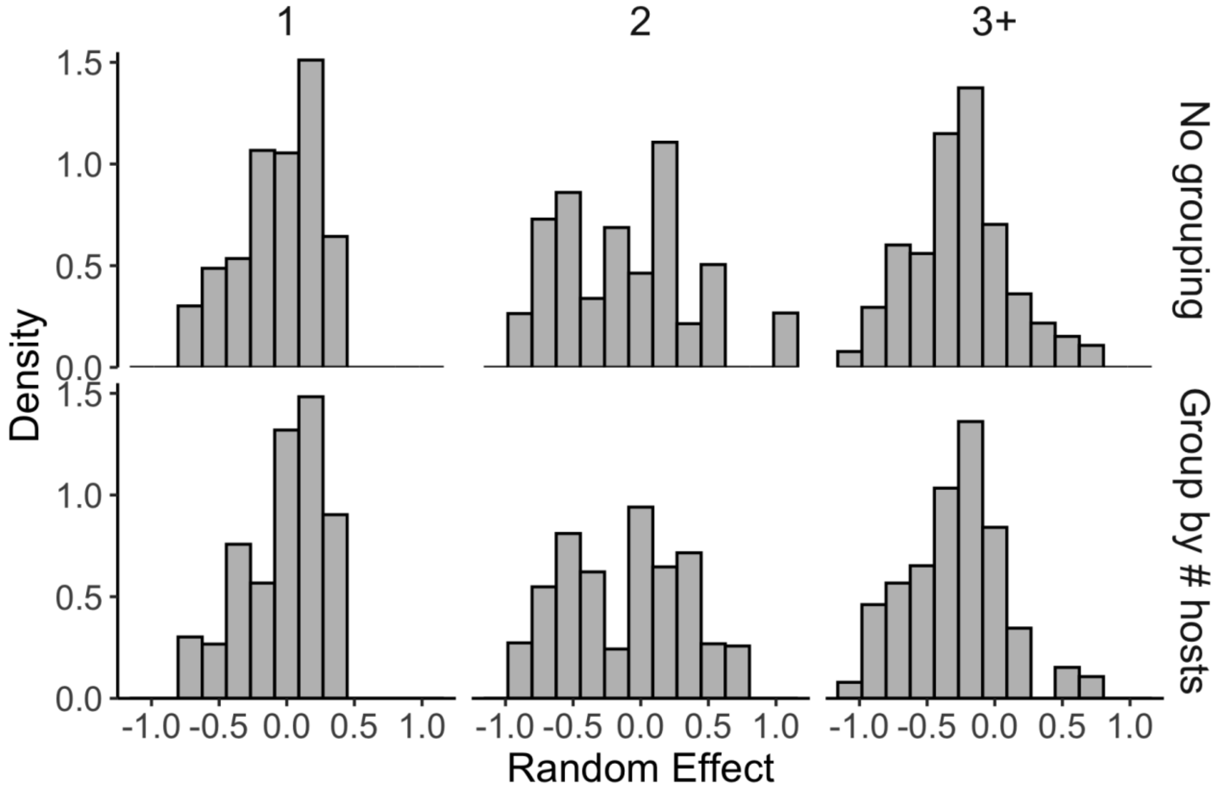

Supplementary Information Figure S6. Diagnostic plots of best-fitting model relating parasite counts to year. Left panel depicts expected (theoretical) versus observed scaled residuals, where each point is an observation and the red line indicates observed = expected. Right panel depicts the scaled residuals as a function of rank-transformed model prediction. The horizontal red line at 0.5 is the expectation, the curved red line is a spline-fit. Asterisks indicate data points deemed to be outliers, based on 250 simulations of the data from the fitted model (i.e., these points fell outside of the prediction envelope).

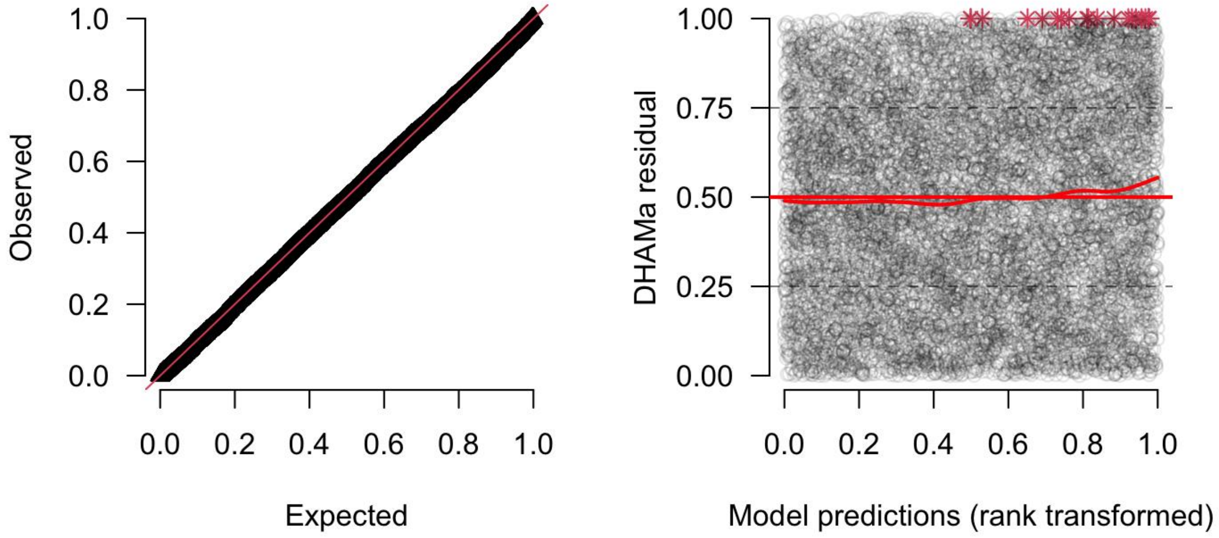

Supplementary Information Figure S7. Diagnostic plots of best-fitting model relating parasite counts to temperature. Left panel depicts expected (theoretical) versus observed scaled residuals, where each point is an observation and the red line indicates observed = expected. Right panel depicts the scaled residuals as a function of rank-transformed model prediction. The horizontal red line at 0.5 is the expectation, the curved red line is a spline-fit. Asterisks indicate data points deemed to be outliers, based on 250 simulations of the data from the fitted model (i.e., these points fell outside of the prediction envelope).

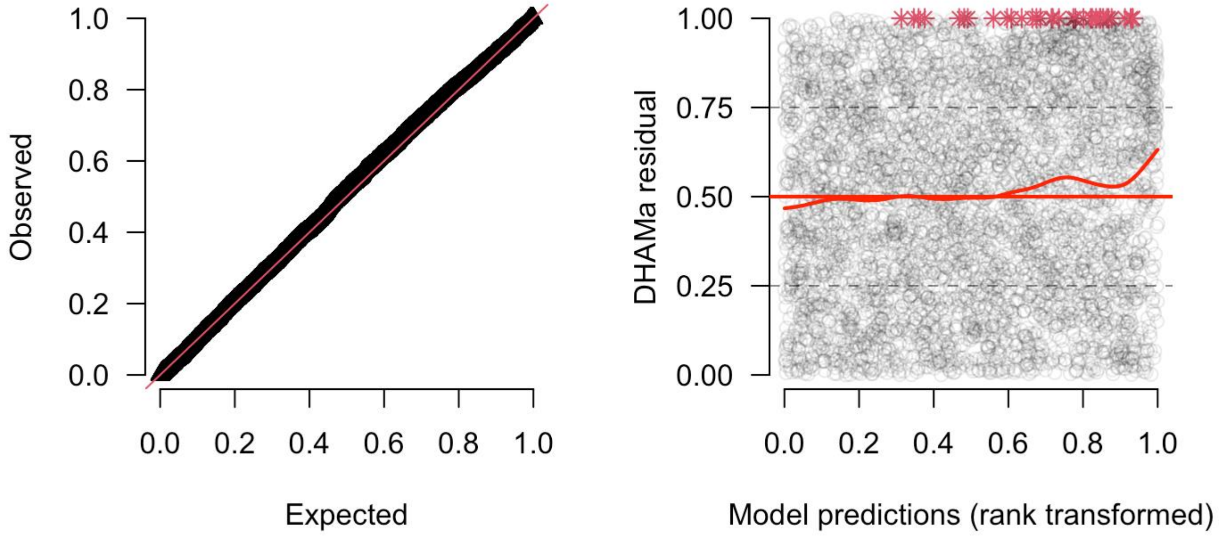

References

Brandenberger, J. M. *et al.* Reconstructing trends in hypoxia using multiple paleoecological indicators recorded in sediment cores from Puget Sound, WA. *National Oceanic and Atmospheric Administration, Silver Spring, MD* (2008).

Froese, R. and D. Pauly. Editors. FishBase. World Wide Web electronic publication. www.fishbase.org. (2022)

Cheung, W. W., T. J. Pitcher, and D. Pauly. A fuzzy logic expert system to estimate intrinsic extinction vulnerabilities of marine fishes to fishing. *Biol. Cons.* **124**, 97–111. (2005).

Hartig, F. DHARMa: residual diagnostics for hierarchical (multi-level/mixed) regression models. R package version 0. 3 3. (2020).
